# Leptin and smoking cessation: secondary analyses of a randomized controlled trial assessing physical activity as an aid for smoking cessation

**DOI:** 10.1186/1471-2458-14-911

**Published:** 2014-09-03

**Authors:** Semira Gonseth, Isabella Locatelli, Raphaël Bize, Sébastien Nusslé, Carole Clair, François Pralong, Jacques Cornuz

**Affiliations:** Department of Epidemiology and Biostatistics, University of California San Francisco, 1450 3rd Street, San Francisco, CA USA; Institute of Social and Preventive Medicine, University of Lausanne’s Hospital, 1010 Lausanne, Switzerland; Department of Ambulatory Care and Community Medicine, University of Lausanne, 1011 Lausanne, Switzerland; Department of Environmental Science, Policy & Management, University of California, Mulford Hall, Berkeley, CA USA; Department of Endocrinology, Diabetology and Metabolism, University of Lausanne, 1011 Lausanne, Switzerland

**Keywords:** Smoking, Smoking cessation, Physical activity, Leptin, Weight gain

## Abstract

**Background:**

Smokers have a lower body weight compared to non-smokers. Smoking cessation is associated with weight gain in most cases. A hormonal mechanism of action might be implicated in weight variations related to smoking, and leptin might be implicated. We made secondary analyses of an RCT, with a hypothesis-free exploratory approach to study the dynamic of leptin following smoking cessation.

**Methods:**

We measured serum leptin levels among 271 sedentary smokers willing to quit who participated in a randomized controlled trial assessing a 9-week moderate-intensity physical activity intervention as an aid for smoking cessation. We adjusted leptin for body fat levels. We performed linear regressions to test for an association between leptin levels and the study group over time.

**Results:**

One year after smoking cessation, the mean serum leptin change was +3.23 mg/l (SD 4.89) in the control group and +1.25 mg/l (SD 4.86) in the intervention group (*p* of the difference < 0.05). When adjusted for body fat levels, leptin was higher in the control group than in the intervention group (*p* of the difference < 0.01). The mean weight gain was +2.91 (SD 6.66) Kg in the intervention and +3.33 (SD 4.47) Kg in the control groups, respectively (*p* not significant).

**Conclusions:**

Serum leptin levels significantly increased after smoking cessation, in spite of substantial weight gain. The leptin dynamic might be different in chronic tobacco users who quit smoking, and physical activity might impact the dynamic of leptin in such a situation.

**Clinical trial registration number:**

NCT00521391

**Electronic supplementary material:**

The online version of this article (doi:10.1186/1471-2458-14-911) contains supplementary material, which is available to authorized users.

## Background

Tobacco use is the world leading cause of preventable death [1]. For many smokers, smoking is viewed as a means for weight control [2–5]. Smoking cessation is indeed associated with around 2.6 Kg of mean weight gain after five years of quitting in men and 3.6 Kg in women [6]. Efficient treatments or preventive interventions to avoid weight gain following smoking cessation are lacking [7]. Nevertheless, there is some promising evidence towards physical activity (PA) being a solution. For instance, a Cochrane review found that one year of regular PA following smoking cessation might help to decrease weight gain [8].

Numerous studies argued that a hormonal mechanism of action might be implicated in post-cessation weight gain, and that leptin may be involved [9–12]. Leptin is a cytokine, which is released from the adipose tissue and acts centrally to suppress food intake and increase the metabolic rate [13]. Leptin decreases the appetite after eating: leptin rises with food intake and modifies the balance between appetite stimulation and inhibition in the hypothalamus, through an action involving neuropeptide Y, leading to a consecutive decrease in food intake [14–16].

Leptin is considered as a potential mediator of weight gain following smoking cessation [10, 17, 18] or as a cause of differences in body weight observed between smokers and nonsmokers [13]. "Crude" serum leptin levels are lower in smokers in comparison with nonsmokers [9, 19–21], however this association disappears when leptin is adjusted for body mass index (BMI) [22]. Only few prospective studies, with small sample sizes and short follow-up have explored the variations of leptin following smoking cessation and their results are contradictory [13, 22–25].

Studies on the effect of physical activity on serum leptin levels are scarce. According to a review, studies with designs that manipulate energy balance showed that serum leptin levels do not change with physical activity unless a marked energy deficit occurs [26, 27]. Published studies about leptin variations and physical activity assessed only intense physical activity, utilizing small samples sizes and short times of intervention.

Given scarce information existing on the biodynamic of leptin during smoking cessation and the plausibility of leptin as a good candidate to explain weight gain following smoking cessation, we attempted to describe the biodynamics of serum leptin levels during smoking cessation. We analyzed also if any variation in insulin secretion and sensitivity impacted the leptin levels–as they are both influenced by smoking cessation and physical activity [28, 29]. We performed secondary analyses of a randomized controlled clinical trial (RCT) assessing moderate physical activity as an aid for smoking cessation in sedentary subjects with an exploratory and hypothesis-free approach.

## Methods

We analyzed data from a population-based randomized controlled trial assessing moderate PA as an aid for smoking cessation. The main outcome of the RCT was the one-year continuous smoking abstinence rate, which was high but not statistically different between the intervention group and the control group (27% versus 29%, p = 0.71). These results are discussed in details elsewhere [30]. Furthermore, the mean weight gain at one-year follow-up was also not significantly different in the two groups [31]. The full inclusion and exclusion criteria, as well as the study design and procedure are described in details elsewhere [30].

### Smoking cessation program

The participants were randomly divided into two groups, "intervention group" and "control group". The sequence of allocation of each group was remotely and randomly generated by a computer, and concealment of allocation was secured by means of sealed envelopes. All participants attended a 9-week program with a weekly 15-minute individual-based smoking cessation intervention combining counseling and prescription of nicotine replacement therapy (16-hour transdermal patches and/or 1- or 2-mg gum, 1-mg lozenge and 10-mg inhaler). This program was based on international guidelines for smoking cessation [32].

Subjects who did not stop smoking at the 5th week after the inclusion visit, or who relapsed, were considered as smokers. Continuous abstinence was defined as not smoking from week 5 until week 52 based on self-report and validated by carbon monoxide measurements (<10 ppm, see below).

Subjects enrolled in the intervention group attended a 9-week weekly 60-minute exercise intervention based on a nationwide implemented moderate-intensity PA program " Allez Hop ! [‘*Let’s go!*’]" with PA-facilitators [33]. The group sessions were divided in three parts: discussion on PA, 45-minute of moderate PA and debriefing. The intervention was targeted to reach a score between 11 (« easy ») and 13 (« somewhat hard ») on the Borg Rating of Percieved Exertion Scale [34]. Participants were encouraged to practice 30-minute of moderate-intensity PA four times per week.

Subjects enrolled in the control group attended a 9-week weekly 60-minute health education program to ensure equal contact conditions. Control subjects participated to group sessions on healthy lifestyle, including lectures, distribution of handouts and discussion about diet, prevention of cardio-vascular disease and cancer, and screening programs for breast and colon cancers. We adaptated a validated program to the Swiss population [35].

Serum fasting leptin, insulin and glucose levels were assessed at baseline, and after 3, 6 and 12 months.

### Data collection

Self-reported smoking abstinence was confirmed by carbon monoxide in exhaled air (<10 ppm), measured with a Micro Smokerlyser® (Bedfont©). PA was quantified with the self-administrated Physical Activity Frequency Questionnaire (PAFQ) [36, 37]. Volume of PA was then calculated in METS*min/week for PA achieved in ≥4 METS, according to American Heart Association [38]. Participants wearing underwear were weighted to the nearest 0.1 kg and their height was measured to the nearest 0.5 cm. BMI was calculated using the formula: weight [Kg]/ height^2^ [cm]. Proportion of lean and fat masses were assessed by leg-to-arm bioelectrical impedance analysis [39]. Plasma leptin was measured by radio-immuno assay, using a kit from Linco Research Inc. (St-Charles, M.O.), in serum samples removed in the morning following an overnight fasting. For costs reasons, leptin was measured only in the first 271 of the total 481 included participants. Percentage of body fat, BMI, and leptin were analyzed as continuous variables. In order to investigate leptin level while controlling for change in adipose tissue, we defined the *ratio leptin/body fat levels* as the ratio between leptin levels [mg/l] and the percentage of body fat [%]. Differences of serum leptin levels between men and women are well described [40]. To estimate insulin secretion and insulin sensitivity among participants, the Homeostasis model assessment for insulin resistance (HOMA-IR) was calculated according to the formula: fasting insulin [mU/l] * fasting glucose [mmol/l]/22.5 [41]. Results are presented for all participants and stratified by sex.

### Statistical analyses

We first performed a comparison between baseline characteristics in the intervention and control group, using two sample t-test for normal distributed variables, non parametric Wilcoxon test for skewed variables and z-test for binary variables. Follow-up time, abstinence duration, total physical activity and proportion of continuous abstinent at 12 months were compared in the two groups using Wilcoxon and z-test. Twelve months changes in weight, BMI, body fat, HOMA-IR, leptin and ratio leptin/body fat (ratio leptin/body fat) were compared using two-sample t-tests.

Changes in leptin variables between the baseline and the subsequent visits were estimated by performing simple linear regressions by study group and by smoking status. We also performed a multivariate linear regression to estimate the impact of potential confounding variables to the association between leptin levels and study group. Individuals with missing data were excluded from these analyses.

We used a polynomial longitudinal model in order to compare the dynamics across the time of the ratio leptin/body fat in the two groups of randomization (the methods are described in the Additional file 1).

We used the R system for all statistical computations and graphics (v 2.11.1, http://www.r-project.org/, function "lme", library "nlme").

## Results

The study population included 271 participants, 136 in the control group and 135 in the intervention group. No statistically significant difference was observed in baseline characteristics between the control and intervention groups (Table 1). The follow-up had a median duration of 55 (IQ = 11-59) weeks and was similar between the intervention and control groups. Of the 271 participants, 81 (29.9%) were continuous abstinent (CO-verified) for the entire duration of the study. During follow-up, participants showed a median duration of abstinence of 11.9 (IQ = 4-52.3) weeks. In the control group, the median abstinence was 16.5 (IQ = 4.5–51.7) weeks, while in the intervention group it was 11.4 (IQ = 3.5 – 53.5) weeks (p of the difference = 0.95). After 12 months of follow-up, the abstinence rate was 50.96% in the control group, and 50.76% in the intervention group (p of the difference = 0.97). The total physical activity in ≥4 METS after 12 months was 879 (203–2240) METS*min/week in the control group, and it was 1642 (758–3084) METS*min/week in the intervention group (p of the difference = 0.01).

**Table 1:** **Baseline characteristics by study group**

	Control (n = 136)	Intervention (n = 135)	p
**Men, No (%)**	81 (59.6)	77 (57.0)	0.674
**Age [years], mean (SD)**	42.7 (9.3)	41.7 (9.7)	0.419
**Number of cig./d, median (IQR)**	25 (20–30)^†^	25 (20–30)	0.697
**No of years of education, mean (SD)**	12.6 (1.8)^‡^	12.5 (1.8)	0.782
**Physical activity in >4 METS, median (IQR)**	936 (188–2047)^*^	866 (190–2403)^**^	0.942
**Weight [Kg], mean (SD)**	70.7 (12.9)	71.8 (15.9)	0.526
**BMI, mean (SD)**	23.8 (3.23)	24.1 (4.12)	0.526
**Percent of body fat, mean (SD)**	25.6 (5.7)	25.8 (7.0)	0.876
**HOMA-IR**	2.22 (1.13)	2.45 (1.39)	0.17
**Leptin [mg/l], median (IQR)**	6.4 (4.2 - 11.7)	7.2 (4.3 - 12.2)	0.376
**Ratio leptin/body fat [mg/l], median (IQR)**	0.26 (0.17 - 0.43)	0.28 (0.19 - 0.48)	0.290

† n = 135 ; ‡ n = 133 ; ^*^n = 115 ; ^**^n = 111

**Figure 1:**
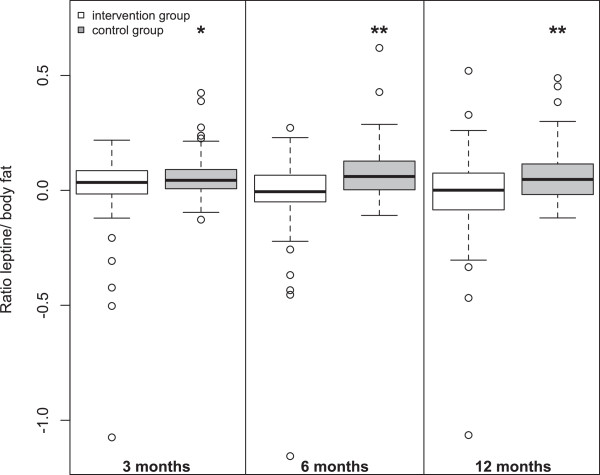
**Ratio leptin/body fat, by study group over the study period (at baseline visit, and at 3, 6 and 12 months).**

Table 2 shows the changes of metabolism-related variables at 3, 6 and 12 months after the baseline visit by study group. The change of leptin was almost significantly higher in the control group at 6 months (p = 0.08), and significantly higher at 12 months (p = 0.014). The change of the ratio leptin/body fat was higher in the control group at each visit, and it was strongly significant at 6 and 12 months of follow-up (p = 0.024, 0.001 and 0.008, at 3, 6 and 12 months, respectively), as illustrated in the Figure 1. The other variables did not show any significant change between the groups. Table 3 shows the same variables by smoking status, independently of the study groups. No significant association was observed.

**Table 2:** **Change in follow-up variables at 3, 6, and 12 months after the baseline visit among all participants, by study group**

	3 months			6 months			12 months		
	Intervention	Control	p ^¶^	Intervention	Control	p	Intervention	Control	p
**Body weight [Kg]**	2.31 (2.72)	2.15 (2.48)	0.665	2.84 (5.99)	3.02 (3.51)	0.822	2.91 (6.66)	3.33 (4.47)	0.633
**BMI [kg/m2]**	0.8 (0.91)	0.73 (0.89)	0.57	1.01 (1.83)	1.03 (1.21)	0.928	1 (1.98)	1.14 (1.53)	0.624
**Body fat [%]**	1.76 (4.43)	1.09 (2.13)	0.269	2.53 (5.75)	1.86 (3.19)	0.477	2.49 (5.24)	2.53 (2.95)	0.957
**HOMA-IR**	0.1 (1.16)	0.04 (1.22)	0.735	0.33 (1.08)	0.24 (1.03)	0.596	0.67 (1.56)	0.78 (1.92)	0.731
**Fasting leptin [ug/l]**	1.78 (3.79)	2.37 (3.61)	0.273	1.73 (5.77)	3.2 (4.42)	0.08	1.25 (4.86)	3.23 (4.89)	0.014*
**Ratio leptin/body fat**	0 (0.19)	0.06 (0.1)	0.024*	-0.04 (0.23)	0.08 (0.12)	0.001**	-0.02 (0.22)	0.07 (0.12)	0.008**

BMI: Body Mass Index; HOMA-IR: homeostasis model assessment for insulin resistance.

¶ p-value, assessed by univariate linear regression analysis.

Values are expressed in means (standard deviation).

* p <0.05;**p <0.01.

**Table 3:** **Change in follow-up variables at 3, 6, and 12 months after the baseline visit among all participants, by smoking status (continuous abstinence vs. relapse)**

	3 months			6 months			12 months		
	Smoking	Abstinence	p	Smoking	Abstinence	p	Smoking	Abstinence	p
**Body weight [Kg]**	2.25 (2.35)	1.99 (2.83)	0.584	3.05 (4)	2.45 (6.15)	0.599	2.45 (3.64)	3.2 (7.42)	0.520
**BMI [kg/m2]**	0.78 (0.89)	0.68 (0.95)	0.539	1.08 (1.44)	0.86 (1.87)	0.535	0.84 (1.2)	1.1 (2.29)	0.468
**Body fat [%]**	1.49 (3.19)	1.23 (4)	0.784	2.18 (3.1)	2.09 (6.01)	0.955	2.5 (2.84)	2.57 (5.47)	0.948
**HOMA-IR**	0.1 (1.56)	0.03 (1.03)	0.763	0.27 (1.2)	0.27 (0.93)	0.977	0.99 (2.32)	0.83 (1.54)	0.681
**Fasting leptin [ug/l]**	1.8 (4.3)	2.03 (3.67)	0.734	1.88 (6.82)	2.63 (4.59)	0.505	1.83 (4.14)	2.56 (5.44)	0.455
**Ratio leptin/body fat**	0 (0.15)	0.03 (0.17)	0.513	-0.02 (0.18)	0.02 (0.22)	0.529	0 (0.11)	0.03 (0.24)	0.564

Values are expressed in means (standard deviation).

In the multivariate linear regression, the change of the ratio leptin/body fat was significantly different according to the study group, being lower in the intervention group at each visit (Table 4). Fasting insulin and glucose levels did not impact this association (no significant p values, expected for insulin at 6 months, where p = 0.011) neither did the smoking status.

**Table 4:** **Multivariate linear regression analysis of the changes in the ratio leptin/body fat at 3, 6 and 12 months after the baseline visit, respectively**

	3 months (n = 124)		6 months (n = 95)		12 months (n = 101)	
	RC	p	RC	p	RC	p
**Intercept**	-0.084	0.469	-0.072	0.609	0.145	0.579
**Intervention**	-0.238	0.040*	-0.426	0.002**	-0.115	0.030*
**Smoking status**	-0.062	0.602	-0.122	0.417	-0.018	0.749
**Fasting insulin [mU/l]**	0.204	0.084	0.466	0.008**	0.005	0.174
**Fasting glucose [mmol/l]**	0.091	0.429	-0.057	0.705	-0.023	0.655

RC : regression coefficient.

* p <0.05;**p <0.01.

Applying a longitudinal model, we found that the mean ratio leptin/body fat increased in the first 35 weeks, and decreased thereafter, following a quadratic curve (p < 0.001 for both the linear and quadratic term, Additional file 2: Figure S1). We also found a significant interaction between the study group and time (p = 0.04), attesting a different pattern of ratio leptin/body fat over the follow up for the control and the intervention group. Namely, the intervention group showed an attenuated dynamics of the ratio leptin/body fat across the time, with a maximum gain of 0.04 ml/l instead of 0.07 ml/l at 35 weeks, and a final gain of 0.02 ml/l instead of 0.04 ml/l at the median follow-up duration of 55 weeks.

Finally, with a piecewise longitudinal model applied to the 234 participants who stopped smoking at least once, we could give a separate estimate of the ratio leptin/body fat change during abstinence and relapse episodes during follow-up (Additional file 2: Figure S2). Physical activity still had an influence on leptin dynamics during abstinence periods, with a more important increase for the control than the intervention group (p = 0.03). The model also showed a significant effect of sex on ratio leptin/body fat initial levels and dynamics across time: women had a baseline ratio leptin/body fat level significantly larger than men (0.48 ml/l vs. 0.20 ml/l, p < 0.001), and a more important dynamics over the follow-up. Relapse to smoking modified the quadratic pattern of the adjusted leptin, leading to an abrupt linear decrease of the mean adjusted leptin when the relapse episode started (p < 0.001).

## Discussion

We found that the levels of leptin significantly increased during the study period in all study participants, independently of their study group. The increase of serum leptin was the greatest (+5.27 mg/l, SD 5.59) in continuous abstinent participants from the control group, which constitutes an increase of more than 82% compared with the mean control group’s baseline leptin levels. The levels of adjusted for body fat leptin increased after 12 months in the control group, but not in the intervention. In addition, the participants in both groups gained substantial weight (e.g., between +6.04 Kg (SD 3.87) in control abstinent group and +4.66 Kg (SD 4.18) in intervention abstinent group over 12 months). Our findings bring more solid evidence of the post-cessation leptin dynamic than what was previously found in smaller studies with shorter follow-up on that same subject [13, 22–25].

It is probable that leptin levels increased after smoking cessation because the study’s participants increased their food intake. Indeed, an increase in food intake has been widely observed during smoking cessation [42]. It is caused, most probably, by a compensatory eating behavior in response to the tobacco withdrawal [43]. According to previous studies in nonsmokers, leptin increases with an increase in food intake. For instance, in a study conducted by Weigle et al., overeating rapidly increased serum leptin levels by about 40 percent [44]. Moreover, the observed increase of leptin was independent of any major influence of insulin secretion or sensitivity.

However, when strictly considering the leptin pathway, our findings were counter-intuitive. Indeed, the expected effects of an increase of leptin levels would be a consecutive decrease in the appetite, thus in food intake. No weight gain should thus arise in such a situation [45], unlike what we observed in our study. Several hypotheses can be made to explain this apparent contradiction. For instance, the participants might have been presenting a resistance to leptin, i.e. a rise in leptin was not producing a decrease in appetite (and thus in food intake and body weight) as expected. Chronic tobacco use might induce such a leptin resistance, in a similar way that it is known to produce an insulin resistance, as described by Willi et al. [46]. At this stage, the mechanisms of action of such a metabolic effect of smoking on leptin could only be hypothesized. For instance, a chronic inflammation induced by smoking metabolites could damage the leptin receptors. Other reasons for these apparent contradictory results might involve other hormones or pathways, like insulin for instance. Moreover, it is possible that the increased eating behavior due to the tobacco withdrawal is so strong that is not influenced by the action of leptin. Further research on this subject is needed.

In addition, moderate physical activity seemed to attenuate the increase in serum leptin levels after smoking cessation. Indeed, we found that the participants in the intervention group, who underwent a 9-week smoking cessation program, consisting of 60 minutes per week of moderate physical activity, had a lower increase in serum leptin levels after one year of follow-up than the control group. It is interesting to note that we did not see, as theoretically expected, an increase in weight gain in the intervention group compared to the control group, despite the fact that the intervention group had lower serum leptin levels. Here again, the underlying mechanisms of action of such an effect remain unknown and can only be hypothesized at this stage. In accordance with our first interpretation, what we might see here could be a beneficial impact of physical activity on the leptin resistance -if existing- and induced by smoking, i.e. physical activity might reduce this leptin resistance. We have already known that physical activity had several beneficial impacts on type 2 diabetes insulin resistance [47], and it could produce a similar action on leptin. Such potential beneficial action of physical activity on leptin resistance, if confirmed, might occur, for instance, through a change in the balance between muscular and fat masses, in favor of muscle.

According to our model, we found that smoking cessation triggered an increase in serum leptin levels in both groups, but physical activity attenuated this increase, leading to substantially recover the initial leptin levels at the end of follow-up. This could be interpreted as a decrease in leptin resistance over time after smoking cessation, similar to a "healing process". This might also reflect that the eating behavior due to the tobacco withdrawal attenuates over time. Incidentally, our model showed that a smoking relapse triggered an abrupt decrease in leptin levels. This fall in leptin could be consecutive to the effect that, when a relapse occurred, smoking acted as a substitute for food, then food intake decreased followed by a decrease in serum leptin levels.

Several limitations of our study have to be mentioned. First, the physical activity program of our study was of very moderate intensity, which probably underestimated the potential effect of physical activity on metabolic homeostasis and was discussed also elsewhere [30]. Second, a vast majority of participants – both in the intervention and in the control group – relapsed at different stages during the study. The longitudinal models we used in the supplementary analyses allowed counteracting this limitation, taking into account information for each participant, independently of the beginning and the duration of her or his smoking abstinence and relapse.

## Conclusions

We found that serum leptin levels significantly increased after smoking cessation, while the study participants gained substantial weight in the same time period. This finding is in discordance with the theoretical effects of leptin. Several hypotheses could explain these findings, including for instance a leptin resistance in smokers. These hypotheses need to be further investigated. We also found that moderate physical activity might have attenuated this potential leptin resistance. This study brings additional arguments towards the hypothesis that leptin would be implicated in weight gain after smoking cessation.

## Electronic supplementary material

Additional file 1: **Supplementary material** [31, 48–54]**.** (PDF 173 KB)

Additional file 2: Figure S1: The ratio leptin/body fat mean pattern over the one year follow up according to the randomization group (mixed effects model). **Figure S2.** The ratio leptin/body fat mean pattern during abstinence and relapse episodes of the follow-up, according to sex and randomization group (adjusted piecewise polynomial longitudinal model). (PPTX 115 KB)
