# Supplementary material for: Leptin and smoking cessation: secondary analyses of a randomized controlled trial assessing physical activity as an aid for smoking cessation
Source: BMC Public Health. 2014 Sep 3;14:911. doi: 10.1186/1471-2458-14-911 (PMC4165916; doi:10.1186/1471-2458-14-911)
Supplement: Supplementary file 1 — Additional file 1: Supplementary material [31, 48–54]. (PDF 173 KB) [file 12889_2013_7035_MOESM1_ESM.pdf]

SUPPLEMENTARY MATERIAL

Methods (supplementary)

Longitudinal models are methods of choice for analyzing data with repeated measures for each participant [42, 43]. These models account for inter-individual variability [44, 45]. They can deal with unbalanced data due to missing values and allow for consideration of time in a continuous scale, which is especially useful when measures are not equally spaced for the different subjects, as in our data [46].

We used a polynomial longitudinal model in order to compare the dynamics across the time of the adjusted leptin levels in the two randomization groups. Ratio leptin/body fat data were log-transformed before entering the model, because of their strong right skewness. We also used a *piecewise* polynomial longitudinal model [47, 48] in order to estimate the effect of smoking status on the difference in leptin pattern between the intervention and control groups. The model was estimated on the 234 participants who stopped smoking at least once (86% of total), and the starting time of the model was set at the first quit date of each participant [31]. Assuming that the ratio leptin/body fat stayed constant between baseline and the first quit date, the baseline ratio leptin/body fat level was used as a proxy of the ratio leptin/body fat at the first quit date. The latter was defined as the mid date between the last visit in the smoking status and the first visit in the status of abstinent; relapse dates and higher order quit dates were defined as the midpoint of two consecutive visits involving a change of status (abstinent to relapse, or relapse to abstinent). Besides the randomization group, the analysis was adjusted for the following baseline characteristics: sex, age, reported CPD at baseline, and number of years of education. Of these, only covariates significantly ($p<0.05$) associated with ratio leptin/body fat levels and/or dynamics were kept.
