# Supplementary figures and images for: Leptin and smoking cessation: secondary analyses of a randomized controlled trial assessing physical activity as an aid for smoking cessation
Source: BMC Public Health. 2014 Sep 3;14:911. doi: 10.1186/1471-2458-14-911 (PMC4165916; doi:10.1186/1471-2458-14-911)

## Slide 1
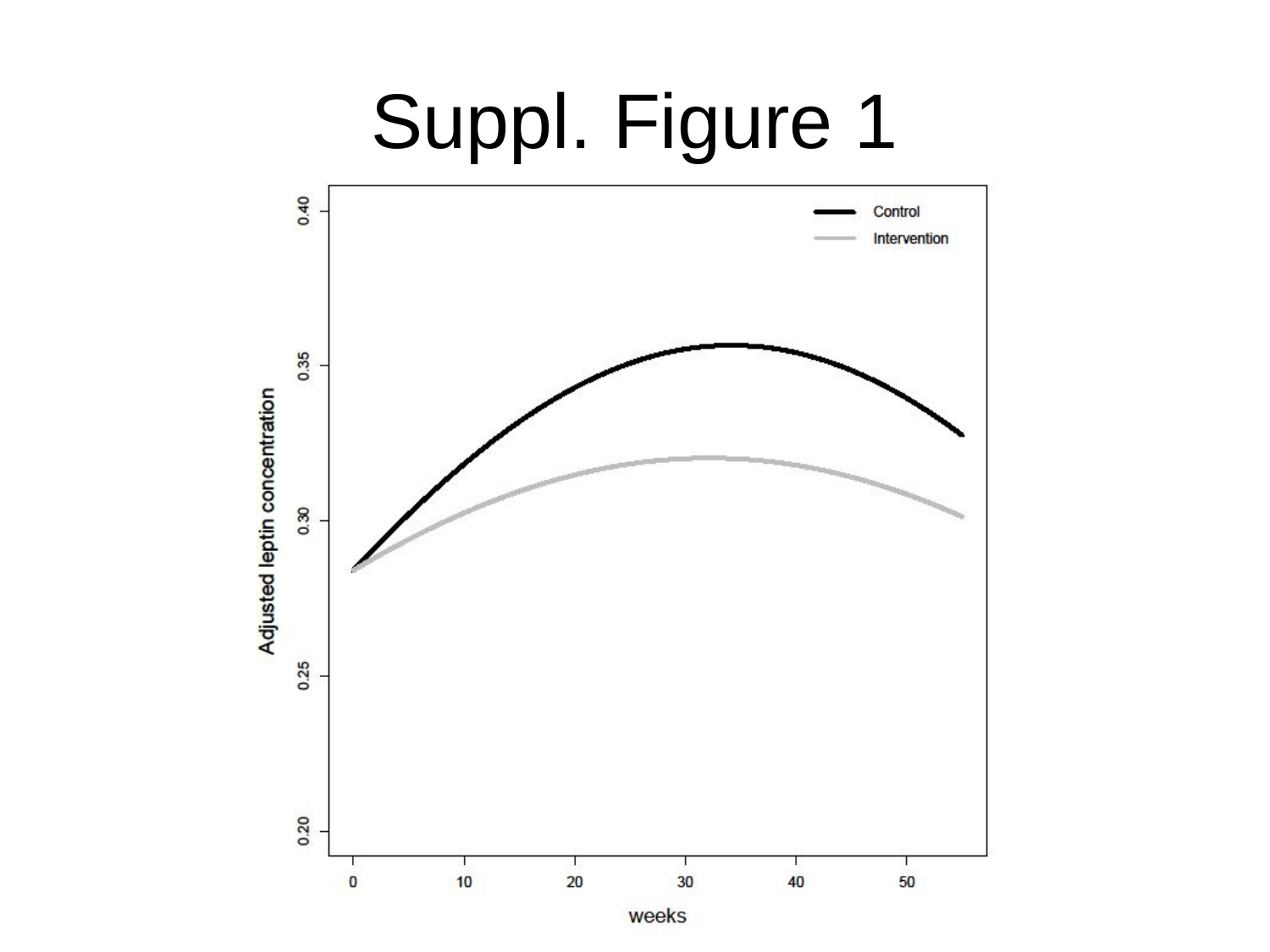

# Suppl. Figure 1

## Slide 2
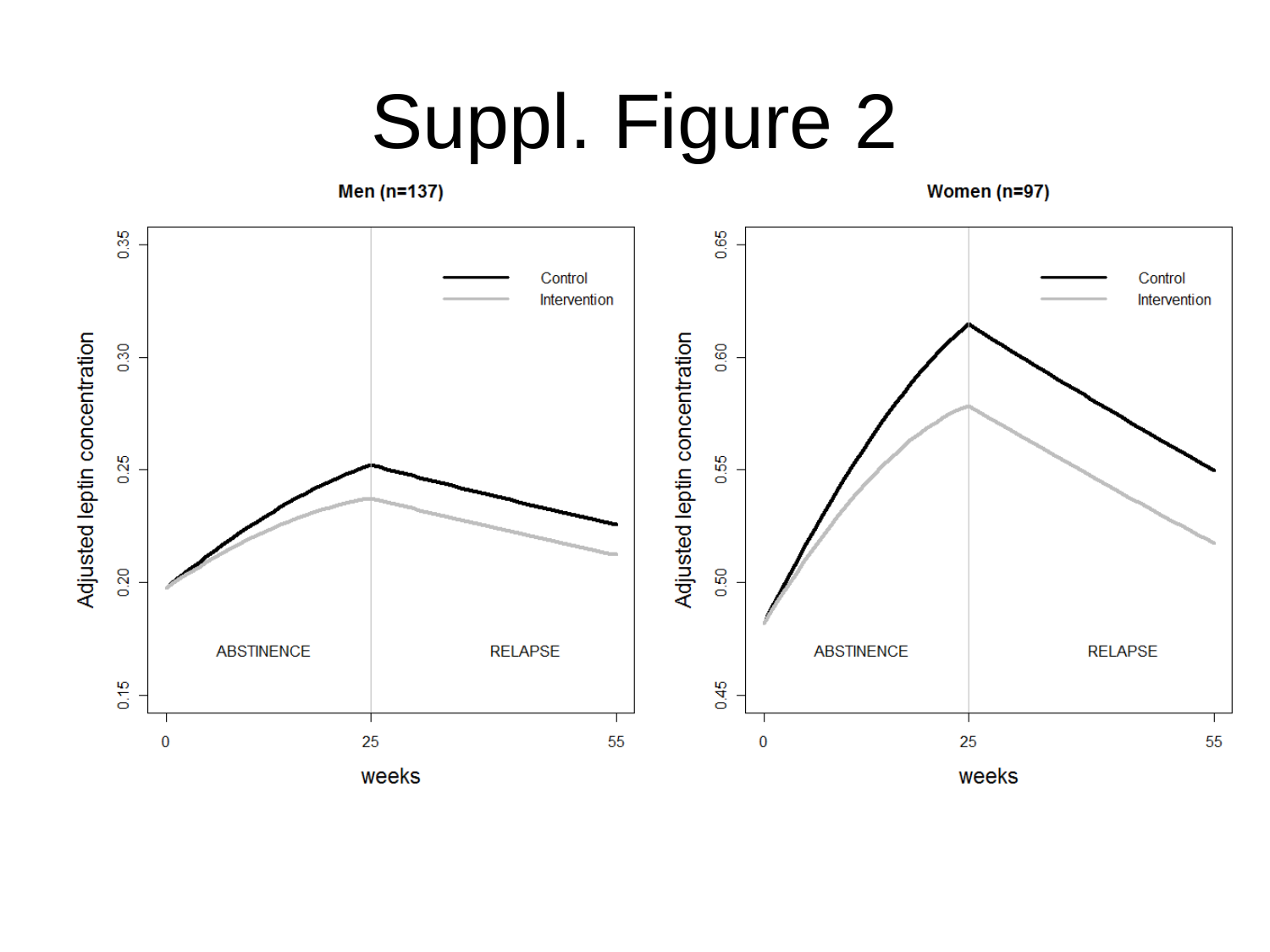

# Suppl. Figure 2

Supplement: Supplementary file 2 — Additional file 2: Figure S1: The ratio leptin/body fat mean pattern over the one year follow up according to the randomization group (mixed effects model). Figure S2. The ratio leptin/body fat mean pattern during abstinence and relapse episodes of the follow-up, according to sex and randomization group (adjusted piecewise polynomial longitudinal model). (PPTX 115 KB) [file 12889_2013_7035_MOESM2_ESM.pptx]
